# Aerosol of Enoximone/Hydroxypropyl-β-Cyclodextrin Inclusion Complex, Biopharmaceutical Evidence for ARDS Applicability

**DOI:** 10.3390/pharmaceutics16091221

**Published:** 2024-09-19

**Authors:** Chiara Migone, Brunella Grassiri, Lucia Vizzoni, Angela Fabiano, Baldassare Ferro, Ylenia Zambito, Anna Maria Piras

**Affiliations:** 1 Department of Pharmacy, University of Pisa, 56126 Pisa, Italy; brunella.grassiri@phd.unipi.it (B.G.); l.vizzoni@student.unisi.it (L.V.); angela.fabiano@unipi.it (A.F.); ylenia.zambito@unipi.it (Y.Z.); anna.piras@unipi.it (A.M.P.); 2 Department of Life Sciences, University of Siena, 53100 Siena, Italy; 3 Anestesia e Rianimazione, Azienda USL Toscana Nord Ovest, 57124 Livorno, Italy; baldo.ferro@gmail.com; 4 Centre for Instrument Sharing of University of Pisa (CISUP), 56126 Pisa, Italy

**Keywords:** ARDS, enoximone, phosphodiesterase-3 inhibitor, inhalation, hydroxypropyl-β-cyclodextrin, pulmonary delivery, lung in vitro model, anti-inflammatory activity, cAMP, oxidative stress protection

## Abstract

Background: Phosphodiesterase (PDE) inhibitors are gaining interest in the context of pulmonary pathologies. In particular, the PDE3 inhibitor enoximone (ENXM) has shown potential relative to the cure of asthma, chronic obstructive pulmonary disease (COPD), and acute respiratory distress syndrome (ARDS). Despite its administration via inhalation being planned for use against COVID-19 related ARDS (C-ARDS), presently, no inhalable medicine containing ENXM is available. Objectives: This study aims to develop a new formulation suitable for pulmonary administration of ENXM. Methods: A solution for nebulization, based on the complex between ENXM and Hydroxypropyl-β-Cyclodextrin (HPβCD) (ENXM/HPβCD) is developed. The obtained solution is characterized in terms of aerodynamic distributions and biopharmaceutical features. Results: The evaluation of the aerosol droplets indicates a good bronchi–lung distribution of the drug. Biological evaluations of the air–liquid interface (ALI) in an in vitro lung cell model demonstrates that ENXM/HPβCD is capable of a local direct effect, increasing intracellular cyclic adenosine monophosphate (cAMP) levels and protecting from oxidative stress. Conclusions: This study offers a promising advance in the optimization of enoximone delivery to the lungs.

## 1. Introduction

Acute respiratory distress syndrome (ARDS) represents the most prevalent form of severe acute hypoxemic respiratory failure [1]. ARDS is an acute and diffuse inflammatory lung injury, presenting as a life-threatening condition in severely ill patients. The onset of ARDS occurs abruptly within seven days of the triggering event. It is characterized by diminished oxygenation, pulmonary infiltrates, bilateral lung infiltrates, and severe, progressive hypoxemia, without any signs of cardiogenic pulmonary edema [2]. Despite extensive research efforts spanning decades, there are still no pharmacological therapies proven to be effective in treating this disease process, leading to persistently high mortality rates [3]. The urgent need for effective ARDS therapies has been highlighted during the COVID-19 pandemic (Covid related-ARDS, CARDS) [4].

Enoximone (ENXM) is a selective phosphodiesterase-3 inhibitor (PDE-3i), marketed as Perfan^®^ (5 mg/mL, drug concentration). Perfan^®^ is an intravenous formulation used in heart failure treatment. Selective PDE-3i block the degradation of cAMP and cGMP in cells where the enzyme is expressed, i.e., in the heart, lungs, platelets, adipose tissue, and inflammatory cell [5]. cAMP and cGMP nucleotides determine the activation of dependent protein kinases such as protein kinase A (PKA) and protein kinase G (PKG). These protein kinases phosphorylate substrates such as ion channels, contractile proteins, and transcription factors, thereby regulating cellular functions [6]. In the myocardium, PDE3 inhibition mainly prevents the degradation of cAMP, causing cardiac inotropy and chronotropy. Furthermore, the reuptake of calcium by the sarcoplasmic reticulum determines the relaxation of the cardiac muscles with a consequent better diastolic function [7]. At the level of the respiratory system, the effects of PDE-3i appear to be manifold, which is why they are considered to be of great interest for the treatment of diseases such as chronic obstructive pulmonary disease (COPD), acute respiratory distress syndrome (ARDS), and asthma [8]. In the lungs, PDE-3i can trigger several pathways acting on pulmonary hypertension, smooth muscle relaxation, vasodilation [9,10,11], as well as on mucociliary clearance modulation [12], inflammation [13], and oxidative stress [14,15]. ENXM has recently been patented in the treatment of atopic immune-relate disorders such as asthma and viral infections such as COVID-19 [16,17]. Beute et al. clearly evidenced the efficacy of ENXM administered intravenously [18] and orally [19]. Indeed, the inhalation route of administration allows for a regional delivery, reducing the incidence of systemic side effects, and generally entails a lower drug dosage. In this context, encouraging preliminary clinical results were observed with Perfan^®^ off-label pulmonary administration [20] in a C-ARDS patient. This initial promising application of inhaled ENXM, combined with compelling clinical evidence from systemically and orally treated patients, opens avenues for the development of ENXM-based pulmonary formulations. However, the aerosolization of the ENXM solution (i.e., Perfan^®^) was hampered by the precipitation of the active principle in the device. Indeed, the precipitation of the drug was not surprising, since it is also stated in the package leaflet of the medicinal product, reporting that ENXM has the tendency to form crystals under certain conditions during handling or storage. In case of nebulization, this crystallization leads to challenges during the administration of the medication. In the abovementioned study, the drug delivery was limited, as the crystals blocked the nebulizer device and hindered a consistent aerosol formation [20]. Hence, overcoming challenges associated with poor delivery dosage, attributed to its tendency to crystallize in the nebulization cup, is imperative.

Cyclodextrins (CDs) are cyclic oligosaccharides comprising (α-1,4)-linked α-D-glucopyranose units, used as functional excipients able to form an inclusion complex with poorly soluble drugs, improving their stability and apparent solubility in aqueous solutions [21]. CDs have been shown to provide many benefits during pulmonary drug delivery, such as improvements to aqueous solubility, systemic absorption, and a better bioavailability of the drugs [22,23]. CDs have been reported to act as potential absorption enhancers during the pulmonary delivery of proteins, such as human growth hormone, insulin, cyclosporine A, etc. [23,24]. In terms of local cytotoxicity, hydroxypropyl-β-cyclodextrin (HPβCD) has been widely investigated, in vitro and in vivo [25,26,27,28], and is considered biocompatible [29]. Additionally, out of the available cyclodextrins, HPβCD has shown optimal tolerability and safety, and, at a 10% *w*/*v* concentration, it did not show any local or systemic toxic effects after nasal administration to rabbits over 3 months [30]. Furthermore, it has been implemented successfully in a clinical study of aerosol administration of budenoside to asthmatic patients, confirming its safety [31].

The aim of this work is to develop an ENXM-based solution to the nebulization issue by exploiting HPβCD as a stabilizing and solubilizing agent to improve drug aerosolization and future pulmonary bioavailability. For this purpose, the ENXM/HPβCD solution is characterized in terms of aerodynamic distributions and biopharmaceutical features.

## 2. Materials and Methods

### 2.1. Materials

ENXM 99.8% was purchased from Tocris (Bristol, UK), Perfan^®^ was purchased from INCA-Pharm (Frosinone, Italy), and Hydroxypropyl-β-cyclodextrin (HPβCD) was kindly provided by Roquette (Whitsett, NC, USA). Dimethilsulfoxyde (DMSO), ethanol, sodium chloride, potassium chloride, acetone, methanol, RPMI amino acid solution, and bicinchoninic acid were purchased from Merck-Sigma (St. Louis, MO, USA), while normal saline solution (NSS) was acquired from Galenica Senese (Siena, Italy). The human immortalized lung adenocarcinoma cell line NCI-H441 (CRM-HTB-174) was obtained from the American Type Culture Collection (ATCC, Manassas, VA, USA). RPMI-1640 Medium (RPMI), L-Glutamine, 1% penicillin/streptomycin, fetal bovine serum (FBS), phosphate-buffered saline free of calcium and magnesium (PBS_A_), dexamethasone 200 nM, and insulin–transferrin–sodium selenite (ITS) were obtained from Merck (St. Louis, MO, USA). Hematoxylin and eosin were purchased from Fluka (Buchs, Switzerland). The cell proliferation reagent (WST-1) and cyclic adenosine monophosphate Elisa assay were provided by Roche diagnostic (Milan, Italy) and Vinci Biochem (Firenze, Italy), respectively.

### 2.2. HPLC Quantification of ENXM

ENXM was detected and quantified using HPLC (Perkin-Elmer, Waltham, MA, USA), equipped with a Series 200 LC-290 AT pump, a Rheodyne injector (20 μL, loop), and a UV–VIS SPD–6 AV detector. The Turbochrom Navigator software (Turbochrom 6.1, Perkin-Elmer, Waltham, MA, USA) and a C-18 reverse phase column (Aeris 3.6 mm, PEPTIDE XB-C18 Å, 250 × 4.6 mm) were used. The mobile phase consisted of methanol/water (60/40). ENXM was detected at 315 nm (RT 6.4 min, calibration curve 0.025–5 µg/mL, R^2^ 0.994, LOD 0.0339 µg/mL, and LOQ 0.0567 µg/mL) when a flow rate of 0.7 mL/min was applied.

### 2.3. Determination of the Stoichiometry of the Inclusion Complex (Job’s Plot)

The stoichiometry of the inclusion complex between ENXM and HPβCD was evaluated with the Job’s plot method [32]. Briefly, two solutions in water were prepared, containing, respectively, (a) ENXM 60.41 µM (starting from a 4 mM stock solution in DMSO) and (b) HPβCD 60.41 µM. With these, two series of dilutions were prepared so that the sum of the molarity of the two remained constant (60.41 µM) (Table S1, supporting information file). The prepared samples were then studied using a UV–VIS spectrometer in a range between 200 nm and 450 nm after 1 h of incubation in a thermostatic shaking bath at 20 °C. The reprocessing of the data was performed by selecting the absorbance at 302 nm, normalized at 450 nm.

### 2.4. Phase Solubility Study

To evaluate the solubilizing properties of HPβCD and determine the association constant (K_a_) of the complex formed by ENXM and HPβCD, a spectrometric titration based on the Benesi–Hildebrand method [33] was conducted. Briefly, two water solutions of 16 µM and 20 mM containing, respectively, ENXM (starting from a 4 mM stock solution in DMSO) and HPβCD were prepared. From these, three series of dilutions were prepared by mixing the ENXM solution with the HPβCD solution, so that the concentration of the guest substance (ENXM) in the solution remained constant (16.1 µM) while the concentration of the host molecule (HPβCD) varied increasingly (Table S2, supporting information file). The series of samples were investigated by UV–VIS spectrometry (operating range 200–450 nm) after 1 h of incubation in a thermostatic shaking bath at 20 °C. The reprocessing of the data was performed by selecting the absorbance at 302 nm, normalized at 450 nm.

### 2.5. Assessment of a Solution for Nebulization Based on ENXM and HPβCD

HPβCD was used as a solubilizing excipient to assess an ENXM-based solution for nebulization. In detail, HPβCD 5% *w*/*v* and an excess of ENXM (0.5 mg/mL) were dissolved in NSS. The suspension was incubated overnight at room temperature and, subsequently, filtered using a 0.45 µm cellulose filter (Whatman, GE Healthcare Life Science) to remove the excess non-complexed enoximone. The preparation was characterized by HPLC to quantify the drug in solution, resulting in 0.3 mg/mL.

### 2.6. Aerodynamic Evaluation of the Nebulized Solution

The ENXM/HPβCD solution was aerosolized using a Benefis pneumatic nebulization device connected to a 12 L/min airflow. As a reference sample, 1:17 Perfan^®^ (corresponding to 0.3 mg/mL of the drug) was used. ENXM was still soluble following the Perfan^®^ dilution with NSS. Aerodynamic properties were assessed using a Twin Stage Impinger (TSI), following the European Pharmacopoeia XII ed., 2.9.18 Apparatus A procedure [34], with the upper and lower stages of the TSI containing 7 mL and 30 mL of 50% *v*/*v* ethanol/water solution, respectively. The T-piece junction of the jet nebulizer was connected to the B portion of the impinger. Additionally, a cold trap and a propylene filter (low resistance filter, PARI, Starnberg, Germany) were placed between the TSI and the mechanical pump (Edwards RV5, Irvine, CA, USA), providing a 60 L/min airflow. The pump was activated 10 s before the nebulizer. At the end of the nebulization process, the two solutions of the stages were collected individually. ENXM concentrations in the two stages, and in the nebulization cup at the end of the nebulization process, were quantified through HPLC. The amount of drug quantified for each stage of the apparatus was expressed as a percentage of the total amount of the collected active substance. Two nebulization durations were implemented, i.e., 1 min and 3 min, respectively. The undelivered samples left in the nebulizer were withdrawn and evaluated by optical microscopy, while crystal dimensions were quantified using ImageJ software 1.50i (Rasband NIH, Bethesda, MD, USA). Each nebulization was conducted out at least in triplicates.

### 2.7. Biological Investigations

The NCI-H441 cell line was employed for biological investigations in vitro and propagated following the supplier’s indications: RPMI-1640 medium with 10% FBS, and 1% pen/strep at 37 °C in a 5% CO_2_ atmosphere.

### 2.8. Cell Viability Studies by WST-1 Assay

NCI-H441 cells were seeded in 96-well plates (4 × 10^4^ cells/well) for the analysis of cell viability after 4 h. The cells were incubated at 37 °C, under 5% CO_2_, and allowed to proliferate for 24 h. The culture medium was removed and replaced with test samples [4].

To prepare the test samples for analysis, the following solutions were prepared:

- Plain ENXM: a stock solution of ENXM in DMSO (1 mg/mL) was prepared, from which dilutions were made in the RPMI-1640 medium to achieve ENXM concentrations in the range of 0.072–1000 μg/mL. All solutions contained 1% DMSO;
- HPβCD: HPβCD solutions in the RPMI-1640 medium, ranging from 0.01% to 10%;
- ENXM/HPβCD solution, with dilutions made in the RPMI-1640 medium, corresponding to 3–150 μg/mL of ENXM and 0.05–2.5% of cyclodextrin, respectively.

After incubation (4 h), the medium was discarded from each well and the cells were washed twice with RPMI-1640. The wells were then filled with RPMI-1640 containing 10% WST-1 reagent solution. After a 4 h incubation, the obtained formazan was measured at 450 nm with a microplate reader (BioTek 800/TS, Thermo Scientific, Walthman, MA, USA). The half-maximal inhibitory concentration (IC_50_) of ENXM/HPβCD was determined.

### 2.9. Direct Nebulization on Differentiated Air–Liquid Interface (ALI) Cell Model

The ALI model was set up as previously described by Vizzoni et al. [4]. Briefly, NCI-H441 cells were seeded (2.5 × 10^3^ cells/well) on Transwell 24-well plates (pore size 0.4 μm, area 0.33 cm^2^). Thereafter, apical and basolateral compartments of the inserts were filled with 100 μL and 600 μL of RPMI-1640, respectively. A total of 48 h post-seeding, the medium was replaced with the RPMI-1640 polarizing medium, which, in addition, contained dexamethasone (200 nM) and the insulin–transferrin–sodium selenite (ITS) supplement. The apical fluid volume was completely removed, leaving the apical surface of the cells exposed to the air (air–liquid culture, ALI). The culture medium was exchanged every two days. Monolayer formation was monitored by transepithelial electrical resistance (TEER) measurement (Voltmeter Millicells-ERS, Millipore, Molsheim, France).

On day 8 post-cell-seeding, the Transwell^®^ inserts containing the differentiated ALI monolayers were placed in a single basolateral chamber, fixed to the bottom of the lower chamber of the TSI [34] and in contact with the connector tube, as the G portion [35] of the TSI was removed. Ultrapure water (7 mL) was placed in the upper chamber. Preliminary tests on monolayer integrity upon nebulization treatment were performed. NSS was nebulized on the monolayer for different time durations, up to 180 s. The integrity of the ALI monolayer was evaluated by TEER measurements before and after the treatments, as well as by hematoxylin/eosin staining. For hematoxylin/eosin staining, the monolayers were washed with deionized water for 5 min. Hematoxylin (0.7% *w*/*v*, sodium iodate, aluminium ammonium sulphate, 12 H_2_O) was then added and incubated for 5 min. Samples were washed and the cytoplasm stained with eosin (0.5% *w*/*v* in acidified 90% ethanol) and incubated for 2 min.

The ENXM/HPβCD solution was nebulized for 30 s, corresponding to 1.5 μg of drug impacting the ALI monolayer. NSS was nebulized as a control sample. Reference drug samples (1.5 μg) were also tested, namely plain ENXM (10 μL of ENXM suspension 150 μg/mL in NSS) was either added directly onto the ALI monolayer or added to the monolayer pretreated with the aerosolized NSS (impacting NSS + plain ENXM). After treatment, the inserts with the ALI monolayers were placed into clean basolateral chambers filled with a medium, then placed in the cell incubator and tested for both drug permeation and drug activity, as reported below.

### 2.10. Drug Effect: Augmentation of Intracellular cAMP Level and Protection from Induced Oxidative Stress

After 1 h of incubation, the elevation in intracellular cAMP levels resulting from phosphodiesterase (PDE) inhibition was quantified using an ELISA assay (Vinci Biochem, Firenze, Italy). The values obtained were normalized in relation to the total protein concentration for each sample which was determined using the bicinchoninic acid assay (BCA). Samples of HPβCD only (0.25 mg corresponding to the amount of CD for 1.5 μg of drug in the ENXM/HPβCD sample) were also tested to exclude any alteration of cAMP levels due to the excipient. The protective mechanism against oxidative stress was also explored. After 1 h of sample pretreatment, cells underwent a 1 h treatment with 1 μM H_2_O_2_ to induce oxidative damage. Following the exposure period, cellular viability was evaluated through the WST-1 assay.

### 2.11. ENXM Permeation through the ALI Monolayer

ENXM permeation through ALI monolayers was performed. At predetermined time intervals (30 min, 60 min, 90 min, 120 min), a volume of 300 μL was withdrawn from the basolateral chamber and subsequently replaced with fresh medium. The quantification of drug permeation in the samples was conducted using HPLC. Data were processed as cumulative plots against time. The experimental procedure was repeated six times (*n* = 6) to ensure statistical robustness and reliability.

### 2.12. Statistical Analysis

The differences between results were assessed for statistical significance using a two-sample Student’s *t*-test in GraphPad Prism software (version 10) program (GraphPad, Boston, MA, USA). 

In all statistical analyses, *p* < 0.05, 0.001 < *p* < 0.01, and *p* < 0.001 were considered significant, very significant, and extremely significant, respectively.

## 3. Results

### 3.1. Phase Solubility Study

The complex formation between ENXM and HPβCD was investigated. A Job’s plot was built on the difference between ENXM absorbance values with and without HPβCD (ΔA). The plot displays the variation of ΔA∙R vs. R, with R representing the drug molar fraction, calculated as [ENXM]/([ENXM] + [HPβCD]). The abscissa value corresponding to the curve maximum represents the complex stoichiometry as 1:2, 1:1, or 2:1, with R = 0.33, R = 0.5, or R = 0.66, respectively [32].

**Figure 1:**
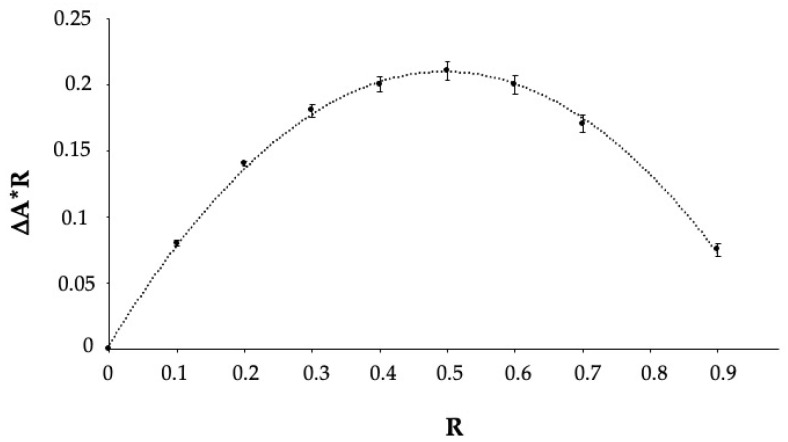
Job’s plot representation of the ENXM/HPβCD complex. ΔA stands for the difference between absorbance values. The ENXM molar fraction value (R) at the maximum of the curve is 0.5.

The maximum obtained R value was 0.5 (Figure 1), corresponding to the 1:1 complex stoichiometry of ENXM/HPβCD.

After establishing the complex stoichiometry, the association constant determined through the modified Benesi–Hildebrand method [33] was estimated at 359 M^−1^. Therefore, HPβCD demonstrated that it was able to efficiently form an inclusion complex with ENXM, increasing its apparent solubility.

The formulation of the ENXM/HPβCD solution for nebulization was set to 5% HPβCD in agreement with the EMA 2017 document [32], asserting that less than 10% HPβCD solutions do not induce tissue damage in nasal and pulmonary products. The applied complexing conditions (incubation time and temperature), followed by the removal of the insoluble excess ENXM, led to a stable solution, since any physical change (opalescence or loss of drug solubility) was observed upon solution storage at RT.

### 3.2. Aerodynamic Evaluation of the Nebulized Solution

A twin stage impinger (TSI) was implemented for the evaluation of the aerodynamic distribution of the aerosol droplets. At a flow rate of 60 L/min, under Ph. Eur. setting [34], the droplets with aerodynamic diameters of more than 6.4 µm were deposited in the first stage of the TSI, while smaller particles were deposited in the second stage. The latter are considered able to reach the deep lung region. Nebulization of the ENXM/HPβCD formulation with a drug concentration of 0.3 mg/mL was performed using the Benefis jet nebulizer for either 1 min or 3 min. As a comparison, the nebulization of 1:17 diluted Perfan^®^ (corresponding to 0.3 mg/mL of drug) was performed. Perfan^®^ dilution was adopted because Perfan^®^ is the only medicine available on the market in Italy containing solubilized enoximone, and because enoximone is not soluble in NSS alone. Each portion of the instrument is designed to accommodate aerosol droplets according to their aerodynamic diameter [34]. The percentages of deposition in each stage are reported in Table 1. As expected, in the case of the 1:17 Perfan^®^, the formation of crystal precipitates was observed in the nebulizer, after both the 1 min and the 3 min nebulization durations. Conversely, the nebulization of the ENXM/HPβCD complex was effective in avoiding the formation of any precipitate. The dimensions of the crystals formed by the 1:17 diluted Perfan^®^ solution were evaluated by optical microscopy. The crystals were 17.0 ± 3.5 µm long and 11.0 ± 2.8 µm wide after 1 min, and 18.0 ± 3.9 µm long and 11.0 ± 2.7 µm wide after 3 min (Table 1). Their formation hampered the aerosolization of the active, resulting in a half-delivered dose compared to the ENXM/HPβCD samples. The jet nebulization of the complex exhibited a better aerodynamic distribution compared to the Perfan^®^ aerosolization, improving the percentage of the drug in the second stage of the TSI. As already noticed, the nebulization of the diluted Perfan^®^ resulted in a poorly delivered dose, with most of the ENXM recovered in the first stage of the TSI (71% and 60% for one and three minutes of aerosolization time, respectively). With the use of ENXM/HPβCD samples, a better distribution of the nebulized droplets was observed, with a rebalancing of the drug percentages for each TSI stage. The increased amount of drug impacting the cell monolayer in the second stage (40% of drug) suggests a better deep lung deposition of the droplets obtained with the cyclodextrin-based formulation.

### 3.3. Cell Viability Evaluation

**Figure 2:**
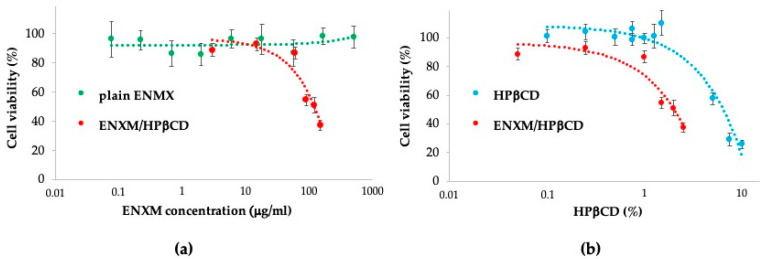
In vitro cytotoxicity of ENXM/HPβCD and its components on NCI-H441 cells. Data expressed as viability percentage for (**a**) ENXM concentration and (**b**) HPβCD concentration (*n* = 6).

A quantitative evaluation of cell viability was performed using the WST-1 assay on the human NCI-H441 cell line. NCI-H441 cells underwent incubation with the ENXM/HPβCD solution, plain ENXM, and HPβCD for 4 h. A high tolerability for the ENXM/HPβCD solution complex was observed (Figure 2). The IC_50_ of ENXM/HPβCD was estimated at 100 μg/mL in terms of ENXM concentration (Figure 2a) and 20 mg/mL in terms of HPβCD (Figure 2b). The addition of HPβCD resulted in enoximone solubilization, enhancing its availability for the cells. This increased availability led to a reduction in cell viability compared to plain ENXM and HPβCD. Despite this effect, the cellular viability values remained considerably elevated, up to 80%, for 80 μg/mL of drug and 1% of HPβCD.

### 3.4. PDE-3 Activity Evaluation on ALI In Vitro Model and Permeation Studies

**Figure 3:**
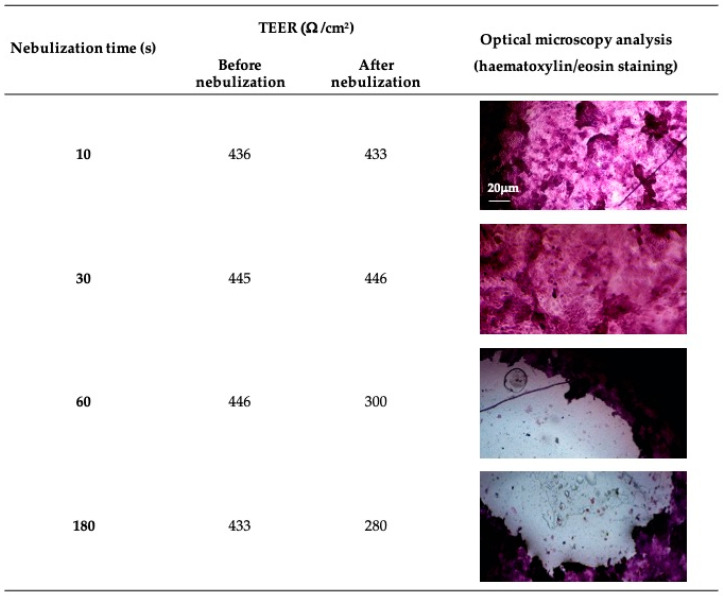
Maintenance of ALI monolayer integrity upon droplet impact under nebulization in the TSI setup. NSS was aerosolized directly onto the NCI-H441 cell monolayer. Transwell^®^ insert NCI-H441 cell monolayers were placed at the base of the lower chamber of a TSI. The nebulization was performed under different duration times. TEER values before and after nebulization and micrographs of NCI-H441 cell monolayers fixed and stained post-nebulization (10× magnification, same scale bar for all micrographs) are presented.

Following cytotoxicity evaluation, drug activity and drug permeation studies on ALI monolayers were conducted. The NCI-H441 cell line cultured at the air–liquid interface mimicked specific phenotypic and functional characteristics of the human alveolar epithelium [4]. The activity and permeation of the drug across the ALI model was assessed by releasing the nebulized drug directly on the cell monolayer placed in the second stage of a TSI. In particular, it accounts for the droplets impacting the deep lung [35]. The maintenance of the monolayer integrity when subjected to droplet impacts in the TSI chamber was first monitored by measuring the TEER before and after the nebulization of NSS (Figure 3). TEER measurements showed the integrity of the cell monolayer after 10 s and 30 s, while, at longer times (60 s and 180 s), TEER values drastically decreased. The data obtained were supported by the micrographs of the cell monolayers obtained by means of hematoxylin/eosin staining. It was possible to note cell detachment from the Transwell^®^ filter relative to the longer nebulization times and the integrity of the cell monolayer in relation to the shorter times (Figure 3). Both TEER values and microscope images evidenced the loss of the integrity of the ALI monolayer after 60 s of nebulization, whereas, after 30 s, the barrier features of the monolayer were still guaranteed.

The activity and permeation studies were thus performed with the highest nebulization time (30 s) that preserved the integrity of the monolayer in order to handle the highest drug dose, in agreement also with the quantification by HPLC assay. The ENXM/HPβCD solution was aerosolized to directly impact the ALI cell monolayer for 30 s (corresponding to 1.5 μg of impacted drug). The plain ENXM (1.5 μg) was used as reference. To exclude any effect due to physical stress, the plain ENXM suspension was also administered after the nebulization of NSS for 30 s. The study did not include the nebulization of the 1:17 Perfan^®^ solution because, in 30 s, it was not delivering enough drug, as evidenced by the aerosolization study. HPβCD alone was also tested.

**Figure 4:**
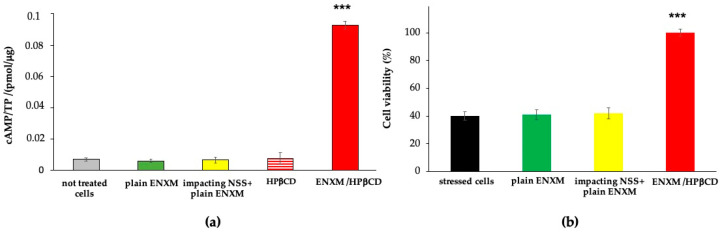
In vitro evaluations of drug activity, drug dose 1.5 μg per ALI monolayer. (**a**) Direct effect of the drug, acting as PDE-3i, thus increasing the intracellular cAMP levels: non-treated cells, drug saline suspension (plain ENXM), plain ENXM administered after NSS nebulization for 30 s (impacting NSS + plain ENMX), HPβCD (0.25 mg), and ENXM/HPβCD were nebulized for 30 s. *** *p* < 0.001 vs. all the other samples. Data were normalized on total protein concentration and expressed as pmol of cAMP per μg of protein. (**b**) Cell viability of H_2_O_2_ stressed cells pretreated with the drug: plain ENXM administered after NSS nebulization for 30 s and ENXM/HPβCD nebulized for 30 s. *** *p* < 0.001 vs. all the other samples.

Following 1 h of incubation, the rise in intracellular cAMP levels induced by PDE-3 inhibition was assessed using an ELISA assay and normalized on total protein content. The results obtained demonstrate that the drug had a direct effect on the increase of intracellular cAMP levels of the NCI-H441 cells. Specifically, when administered in the form of ENXM/HPβCD solution, the levels of cAMP exhibited a noteworthy increase compared to those achieved with plain ENXM under the same dosage of drug treatment (Figure 4a). As observed from the graph, for the ENXM/HPβCD nebulized solution, cAMP levels reached values of 0.090 ± 0.003 pmol/μg, showing significant differences with the values obtained with the plain ENXM (0.006 ± 0.002 pmol/μg) and HPβCD alone (0.007 ± 0.005 pmol/μg). No difference was observed when the plain ENXM was administered directly to the ALI monolayer or after NSS nebulization. These results indicate no contribution due to the mechanical impact onto the cellular monolayer.

Simultaneously, investigations were conducted to elucidate the protective effect against oxidative stress. To induce oxidative damage, cells were subjected to 1 h of incubation with the samples (pretreatment) followed by a 1 h treatment with 1 μM H_2_O_2_. Cellular viability was then assessed using the WST-1 assay, enabling a thorough analysis of potential alterations to cell viability due to oxidative stress induction. The results show the significant protective effect against induced oxidative stress, as depicted in Figure 4b. Cell viability of pretreated ENXM/HPβCD samples reached values of 99%, whereas plain ENXM, either directly added or administered after NSS nebulization, showed values of 41%, comparable to H_2_O_2_ stressed cells.

**Figure 5:**
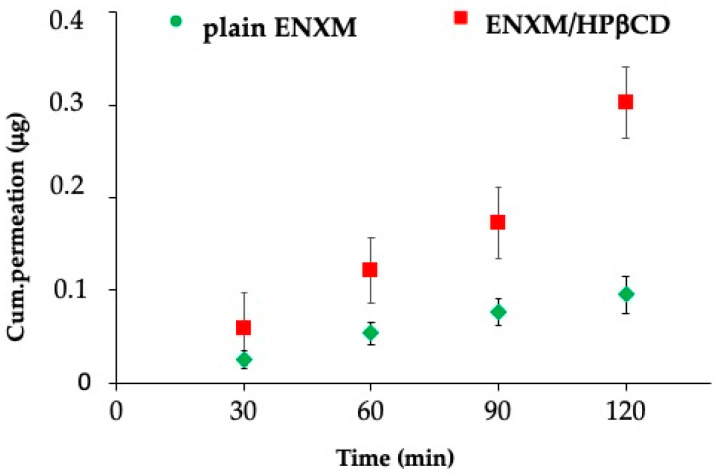
Permeation of the drug across the ALI monolayer of the NCI-H441 cell line, after simulating in vitro the impact of the drug on the deep lung. The cell monolayer was placed in the second stage of a TSI during sample nebulization.

Furthermore, the administration of ENXM through the ENXM/HPβCD solution resulted in a notable 20% increase in drug permeation after two hours, outperforming the comparatively lower 6% permeation achieved when plain ENXM was administered independently (Figure 5).

## 4. Discussion

Phosphodiesterases inhibitors (PDEis) have gained interest in relation to the management of several pulmonary acute inflammations (e.g., ARDS, C-ARDS, interstitial pneumonia) as well as chronic inflammatory conditions (e.g., asthma, COPD, cystic fibrosis, and pulmonary hypertension) [36]. Despite pulmonary administration offering a non-invasive way to deliver drugs directly to the lung of the patients, with a lower required dose compared to the systemic route, no marketed inhalable PDEi drug is presently available. Many inhaled drugs pose formulation and delivery challenges, in part because of poor solubility and stability [37]. In this context, an ENXM inhalable medicine could represent a good opportunity for the treatment of several pulmonary pathologies.

Despite being available as an intravenous formulation, the off-label administration of Perfan^®^ through inhalation has demonstrated the potential of ENXM with regard to improving oxygenation levels and mitigating the requirement for mechanical ventilation in C-ARDS patients [20]. Researchers have also explored the prospects of oral ENXM administration as an adequate alternative to current asthma medications, allowing for the reduction and discontinuation of inhaled steroids and beta 2 agonists in asthmatic children [17]. Moreover, preliminary observations suggest that early intervention with enoximone may help revert respiratory failure as well as avert mechanical ventilation [18]. The pioneering investigation into the beneficial effects of inhaled ENXM [20], coupled with robust clinical evidence supporting its systemic and oral administration [18,19], sparks innovation in the realm of ENXM-based therapies. This prompts a contemplation of the development of pulmonary formulations, envisioning a future where ENXM could be tailored to target respiratory disorders with precision and efficacy.

However, amidst these promising developments, persistent challenges remain. Despite its pharmacological potency, ENXM encounters obstacles with respect to the achievement of optimal delivery and dosage when nebulized as Perfan^®^ [20]. Perfan^®^ nebulization has been shown to result in the formation of solid precipitates in the device under physical pneumatic stress, causing a low nebulization efficacy [38]. In this context, natural cyclodextrins, which are mentioned in the GRAS (“generally recognized as safe”) list of the FDA, could be employed as a stabilizing agent of ENXM due to their ability to form water-soluble complexes with poorly water-soluble drugs [33,39]. In this study, among the different cyclodextrins, HPβCD was chosen for its established safety profile and wide implementation in injectable medicines [40,41], having been already reported in in vivo animal and human evaluations for nasal and lung delivery [30,31]. In the present work, it was firstly demonstrated that HPβCD efficiently formed an inclusion complex with ENXM, significantly enhancing its apparent solubility. The formation of the complex between ENXM and HPβCD was indicative of a 1:1 stoichiometry. The ENXM/HPβCD complex efficiently maintained ENXM in solution, showing a K_a_ of 359 M^−1^. The 5% *w*/*v* concentration of HPβCD was implemented in agreement with EMA indications [30], allowing for an increase of enoximone solubility up to 0.3 mg/mL in NSS. One of the main effects of the ENXM/HPβCD complex was the maintenance of drug solubility during jet nebulization, resulting in a better proportion of drug delivery and better aerodynamic distribution compared to the reference dilution of Perfan^®^. It is thus evident that HPβCD is efficient in keeping ENXM in solution during the nebulization, whereas the excipients found in the composition of Perfan^®^, namely glycerol and ethanol, are not. Indeed, Perfan^®^ is an injectable solution and not a solution for aerosolization; therefore, the mechanical stress is not implied. Considering also the off-label administration of Perfan^®^, the use of NSS as a substitute of pH 12 water (as it is in Perfan^®^) is indeed more acceptable in terms of Ph. Eur. requirements for solutions for inhalation, ruling a pH value within the range of 3–8.5 [34]. Additionally, NSS is preferrable for its humidifying action and decongestant properties, capable of facilitating the elimination of excess mucus and of clearing the upper respiratory tract [42]. An additional confirmation that enoximone is solubilized through complexation with HPβCD is provided by the results of the biological and biopharmaceutical investigations.

Despite the presence of a lower inhibitory concentration respect to plain ENXM, the ENXM/HPβCD could be considered safe and well tolerated by cellular components.

The human lung adenocarcinoma cell line NCI-H441, consisting of distal lung epithelial cells, was cultured at the air–liquid interface in order to recreate the physiological conditions of the respiratory epithelium more accurately compared to traditional submerged cell cultures. When NCI-H441 cells are cultured at the air–liquid interface, they are exposed to both air and culture medium. This setup allowed the cells to differentiate and form a polarized epithelial layer, resembling the structure and function of the alveolar epithelium more closely. This differentiation may lead to the expression of phenotypic and functional characteristics that are relevant to the alveolar epithelium, such as tight junctions, cilia, and the production of surfactant. This model has been applied to the study of various aspects of respiratory physiology, including drug transport, toxicity testing, and infection studies [4,43]. Moreover, the used setting with the ALI monolayer placed in the second chamber of the TSI allowed for the investigation of the effect of the drug carried by the nebulized droplets, mimicking those that would impact the deep lung portion [35]. According to the pharmacopoeia [34], the second chamber of the TSI accurately represents the deep pulmonary pathways. Any findings observed in this chamber after nebulization correspond to the drug passage through the entire respiratory tree, ultimately reaching the deep pulmonary pathways. Being enoximone a PDE3i, the administration of ENXM/HPβCD resulted in elevated intracellular cAMP levels, significantly higher than those associated with the control cells. Additionally, when the plain ENXM was used, no effect was observed, indicating that the drug needs a functional excipient to be bioavailable to the cells. The ability of the drug to elevate intracellular cAMP levels is particularly noteworthy, as cAMP is a key mediator in various cellular processes, including signal transduction and gene expression [44,45]. The mechanism underlying this elevation warrants further investigation, as it may unveil novel insights into the potential applications of the drug. It was also clear that a secondary effect of the increased cAMP levels is cell protection from induced oxidative stress. It is supposed that the maintenance of high intracellular cAMP levels causes an indirect reduction of reactive oxygen species (ROS) production through the nicotinamide adenine dinucleotide phosphate oxidase (NOX) enzymes phosphorylation by PKA [46]. Notably, when compared to the plain ENXM, the ENXM/HPβCD combination demonstrates a significant increase in cell viability under conditions of oxidative challenge. This compelling evidence positions the drug as a promising candidate for therapeutic interventions where cellular resilience to oxidative stress is a critical factor. It is also noteworthy that the use of HPβCD significantly enhanced drug permeation across NCI-H441 cell monolayers. Cell permeation studies revealed that, after 2 h, the permeation of ENXM was approximately 20% when nebulized with the ENXM/HPβCD complex solution, whereas it was only 6% in the case of the reference drug solution. The permeation of enoximone suggests a possible effect of the drug also at the capillary level. Indeed, this is the main effect of the drug when it is administered systemically.

Taken together, the collected data reveal optimal characteristics for the delivery of the drug to the deep lung, offering a promising solution for the lung administration of enoximone. The unique attributes of the discussed drug, encompassing its impact on intracellular cAMP levels and its remarkable protective effect against oxidative stress, position it as a multifaceted therapeutic agent with implications for diverse biomedical contexts. However, it is important to note that the apparent solubility achieved with HPβCD is much higher than the intrinsic drug water solubility but lower than that found in the intravenous product Perfan^®^. The results obtained are promising, particularly in terms of solubility and permeation. However, to ensure that effective therapeutic concentrations reach the lungs within reduced time frames and volumes, an optimized formulation could be suggested to achieve a clinically usable dosage form.

## 5. Conclusions

In conclusion, the formulated inhalation solution based on ENXM and HPβCD demonstrates effective stabilization of ENXM, exhibiting favorable biopharmaceutical properties and making it a promising candidate for ARDS applicability. Differently from the observations made in relation to the direct nebulization of the ENMX containing the marketed solution Perfan^®^, the HPβCD in the ENXM/HPβCD complex is able to efficiently stabilize the drug, preventing its precipitation in solution during nebulization as well as its precipitation in the droplets collected afterward. The assessed formulation shows an improved deposition profile upon nebulization: a two-fold improvement in the delivered dose and a 13% increase in the nebulized drug collected from the second stage of the TSI. Moreover, the use of HPβCD plays a crucial role, as it simplifies the formulation of the water solution, now containing only the drug, HPβCD, and NaCl. In this way, the solution is easy to handle for administration and the ENXM is able to exert its effect on the in vitro lung model, supported by good biopharmaceutical evidence. These features are assessed using an in vitro model which mimics the in vivo administration by directly collecting the nebulized droplets onto ALI cell monolayers placed in the second stage of TSI. The improved drug permeation across the ALI cell monolayers with the ENXM/HPβCD formulation compared to the plain ENXM solution indicates a more effective lung delivery, potentially enhancing the therapeutic outcomes. Additionally, the preservation of drug activity is observed when ENXM/HPβCD is directly nebulized onto the in vitro cell model, resulting in a significant increase in intracellular cAMP levels and offering robust protection against inflammation and oxidative stress. Future research should focus on evaluating the long-term stability of the formulation under various storage conditions and on in vivo studies to further assess the possible clinical applicability of the formulation. This work represents the initial phase of a vast research effort aimed at developing ENXM inhalable medicines.

## Figures and Tables

**Table 1:** Nebulization of the ENXM/HPβCD solution compared to the 1:17 Perfan^®^ for 1 min and 3 min. The amount of drug delivered, size of the formed ENXM crystals, and percentage distribution of the aerosol particles (mean ± SD, *n* = 6) are presented.

Time	Sample	Crystal Size		TSI			
(min)		Length μm ± SD	Width μm ± SD	Delivered mg ± SD	First Stage %	Second Stage %	Filter %
1	ENXM/HPβCD	Absence of crystals	Absence of crystals	0.12 ± 0.03	53 ± 6	35 ± 10	12 ± 7
3	ENXM/HPβCD	Absence of crystals	Absence of crystals	0.14 ± 0.04	47 ± 6	42 ± 6	11 ± 3
1	1:17 Perfan^®^	17 ± 4	11 ± 3	0.05 ± 0.01	71 ± 13	23 ± 7	6 ± 3
3	1:17 Perfan^®^	18 ± 4	11 ± 3	0.07 ± 0.01	60 ± 7	29 ± 10	11 ± 2

## Data Availability

The data presented in this study are available on request from the corresponding author.

## Supplementary Materials

The following supporting information can be downloaded at: https://www.mdpi.com/article/10.3390/pharmaceutics16091221/s1. Table S1. Sample composition for the Job’s Plot analysis, Table S2. Dilutions of enoximone for the association constant determination.
