# Supplementary material for: Aerosol of Enoximone/Hydroxypropyl-β-Cyclodextrin Inclusion Complex, Biopharmaceutical Evidence for ARDS Applicability
Source: Pharmaceutics. 2024 Sep 19;16(9):1221. doi: 10.3390/pharmaceutics16091221 (PMC11435411; doi:10.3390/pharmaceutics16091221)
Supplement: Supplementary file 1 [file pharmaceutics-16-01221-s001.zip › pharmaceutics-3178145-supplementary.pdf]

Article

Aerosol of Enoximone/Hydroxypropyl-β-Cyclodextrin Inclusion Complex, Biopharmaceutical Evidence for ARDS Applicability

Chiara Migone [1,*], Brunella Grassiri [1], Lucia Vizzoni [1,2], Angela Fabiano [1], Baldassare Ferro [3], Ylenia Zambito [1] and Anna Maria Piras [1,4]

[1] Department of Pharmacy, University of Pisa, 56126 Pisa, Italy; brunella.grassiri@phd.unipi.it (B.G.); l.vizzoni@student.unisi.it (L.V.); angela.fabiano@unipi.it (A.F.); ylenia.zambito@unipi.it (Y.Z.); anna.piras@unipi.it (A.M.P.)

[2] Department of Life Sciences, University of Siena, 53100 Siena, Italy

[3] Anestesia e Rianimazione, Azienda USL Toscana Nord Ovest, 57124 Livorno, Italy; baldo.ferro@gmail.com

[4] Centre for Instrument Sharing of University of Pisa (CISUP), 56126 Pisa, Italy

* Correspondence: chiara.migone@unipi.it; Tel.: +39-050-2219614

SUPPLEMENTARY FILE

TABLES

Table S1. Sample composition for the Job's Plot analysis.

Samples	ENXM	HPβCD
	μM	μM
**1**	0.00	60.41
**2**	6.04	54.36
**3**	12.08	42.28
**4**	18.12	36.24
**5**	24.16	30.20
**6**	30.20	24.26
**7**	36.24	18.12
**8**	42.28	12.08
**9**	54.36	6.04
**10**	60.41	0.00

Table S2: Dilutions of enoximone for the association constant determination. Dilutions were carried out starting from stock solutions of ENXM (4µg/ml), HPβCD (420mg/ml) and water.

	**Volume (µl) of the stock solutions**		
**Series**	**ENXM**	**HPβCD**	**H_2O**
**1**	100	0	900
**2**	100	45	855
**3**	100	113	788
**4**	100	225	675
**5**	100	333	567
**6**	100	450	450
**7**	100	563	338
**8**	100	643	257
**9**	100	677	223
